# Hydrogels in Spinal Cord Injury Repair: A Review

**DOI:** 10.3389/fbioe.2022.931800

**Published:** 2022-06-21

**Authors:** Zhenshan Lv, Chao Dong, Tianjiao Zhang, Shaokun Zhang

**Affiliations:** ^1^ The Department of Spinal Surgery, 1st Hospital, Jilin University, Jilin Engineering Research Center for Spine and Spine Cord Injury, Changchun, China; ^2^ Key Laboratory of Pathobiology, Ministry of Education, College of Basic Medical Sciences, Jilin University, Changchun, China; ^3^ Medical Insurance Management Department, China-Japan Union Hospital of Jilin University, Changchun, China

**Keywords:** spinal cord injury, hydrogel, cells, molecular, repair, review

## Abstract

Traffic accidents and falling objects are responsible for most spinal cord injuries (SCIs). SCI is characterized by high disability and tends to occur among the young, seriously affecting patients’ lives and quality of life. The key aims of repairing SCI include preventing secondary nerve injury, inhibiting glial scarring and inflammatory response, and promoting nerve regeneration. Hydrogels have good biocompatibility and degradability, low immunogenicity, and easy-to-adjust mechanical properties. While providing structural scaffolds for tissues, hydrogels can also be used as slow-release carriers in neural tissue engineering to promote cell proliferation, migration, and differentiation, as well as accelerate the repair of damaged tissue. This review discusses the characteristics of hydrogels and their advantages as delivery vehicles, as well as expounds on the progress made in hydrogel therapy (alone or combined with cells and molecules) to repair SCI. In addition, we discuss the prospects of hydrogels in clinical research and provide new ideas for the treatment of SCI.

## 1 Introduction

**FIGURE 1:**
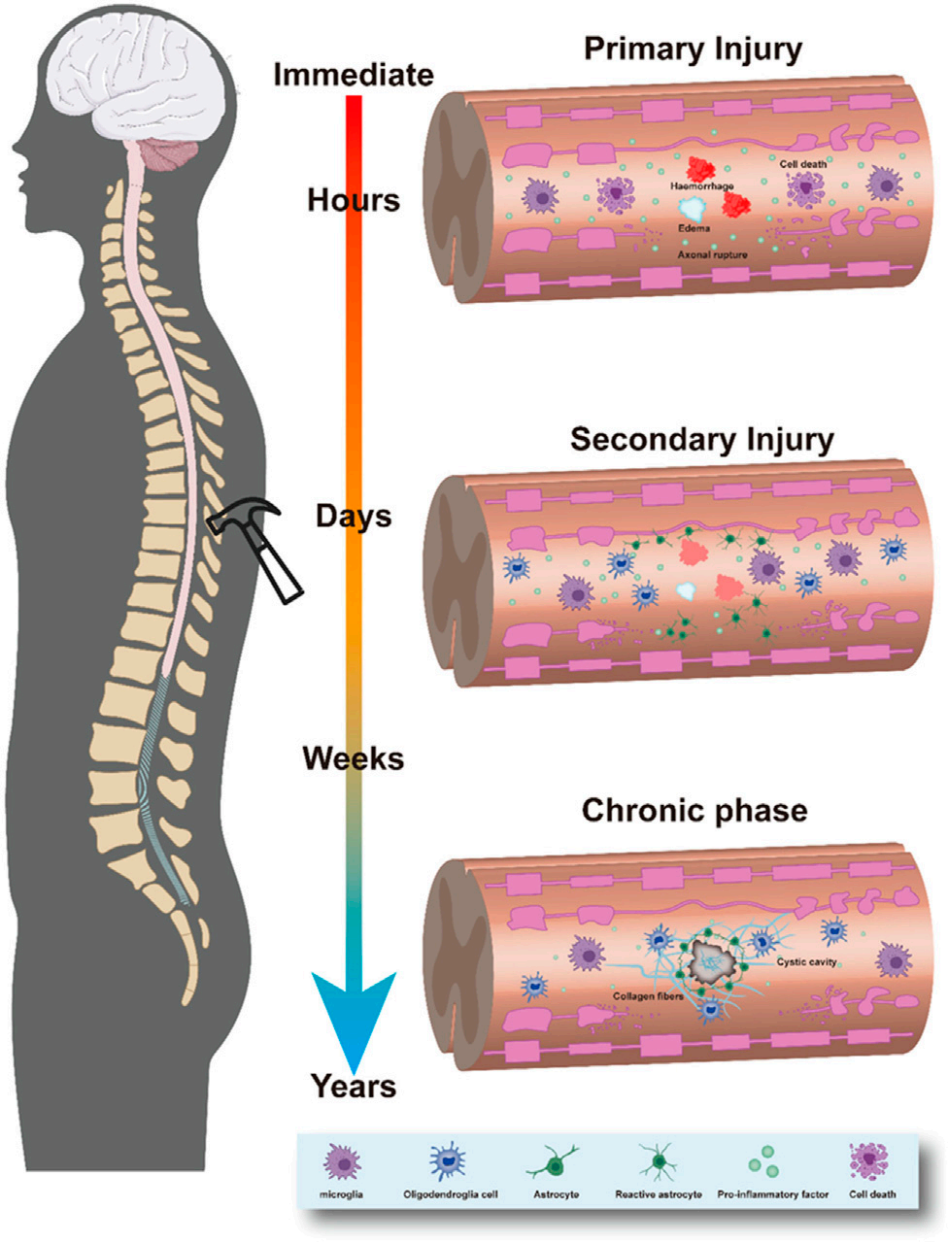
Pathological process of SCI. This process consists of three phases, the primary phase, secondary phase, and chronic phase.

SCI is among the most serious traumas to the nervous system. They are caused mainly by traffic accidents or violence but can also be caused by inflammation and tumors. SCI has high morbidity and disability rates and can lead to the dysfunction of various systems (including the autonomic nervous system) and multiple organs (e.g., those of the respiratory, circulatory, urinary, and digestive systems) (Baptiste and Fehlings, 2006). SCIs can be divided into primary and secondary injuries. Primary SCI is characterized by irreversible damage caused by external forces acting (directly or indirectly) on the spinal cord. Pathophysiological characteristics include edema, cellular damage (Ek et al., 2012; Chen et al., 2021; Song et al., 2021), inflammation, oxidative stress, apoptosis, and necrosis (Moon et al., 2012). Secondary injury refers to spinal cord edema caused by an external force, as well as further damage to the spinal cord caused by spinal cord compression, which may be due to fragmented intervertebral disc tissue, imbalance of gliosis, cytokine release, excessive proliferation of gliocytes, microglia and macrophage accumulation at the injury site, or the formation of astroglial scars, fibrous scars, and syringomyelia (Emmez et al., 2010; Katoh et al., 2019; Onyango et al., 2021) (Figure 1). Secondary injury can further lead to tissue damage and permanent loss of function (Hall and Springer, 2004), seriously affecting nerve repair and complicating synapse reconstruction.

## 2 Current Treatments and Limitations

**FIGURE 2:**
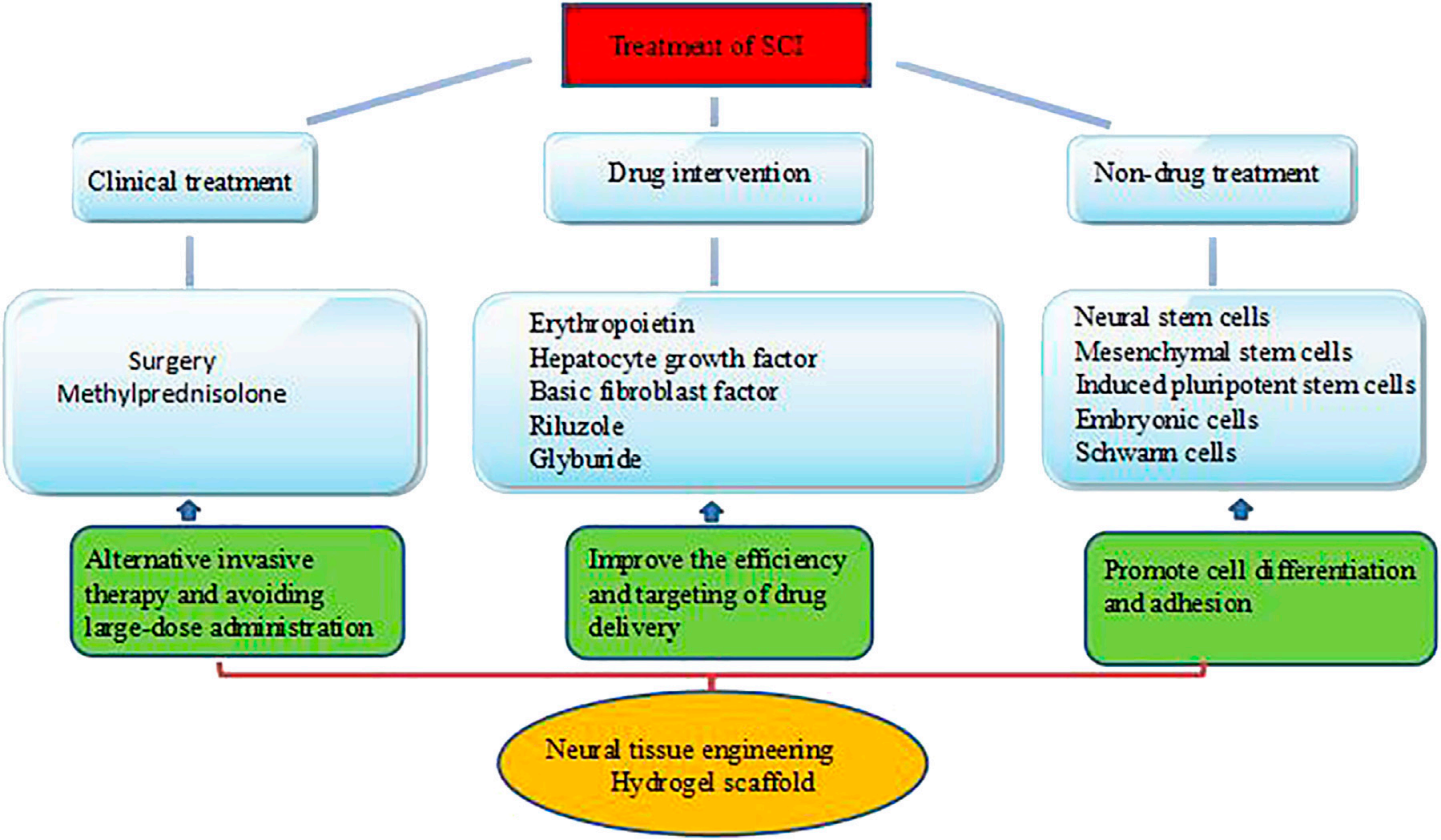
Current treatment and limitations of SCI.

Current treatments for SCI have nine key foci: 1) achieve spinal cord structural reconstruction, 2) inhibit astrocyte scarring, 3) promote nerve cell regeneration, 4) regulate the distribution of the extracellular matrix, 5) repair the microenvironment after injury, 6) remodel damaged neurons and axonal conduction, 7) inhibit secondary injury (Fitch et al., 1999; Choo et al., 2008; Donnelly and Popovich, 2008; Badner et al., 2017), 8) improve the microenvironment around the injury in time (Tuszynski et al., 1996; Kommareddy and Amiji, 2005), and 9) restore the nutrient supply to the spinal cord for axon regeneration (Im et al., 2010; Cregg et al., 2014). SCI treatment primarily includes surgical, pharmacological, and biological approaches (Cox et al., 2015) (Figure 2). The surgical method essentially decompresses the continuously compressed spine, improves the neuroprotection of the SCI, and restores the stability of the damaged spine. However, it is an invasive approach, and there is no uniform standard for the operation time. The main clinical treatments for reducing secondary injury include methylprednisolone, dexamethasone, naloxone, erythropoietin, and neuregulin (Rabchevsky et al., 2011), which improve neurological function in patients by reducing the production of inflammatory substances and inhibiting lipid peroxidation at the injury site. However, intravenous administration of large doses can trigger infection symptoms such as gastrointestinal bleeding and wound infection—such treatments remain controversial and restricted (Silva et al., 2014; Hurlbert et al., 2015). In addition, traditional administration methods, such as oral or intravenous injection (modest doses), are associated with limited crossing of the blood-spinal cord barrier, reducing direct action at the injury site. The complex pathophysiological changes of SCI and the harsh local microenvironment are not conducive to regeneration. Therefore, no single treatment method can repair damaged nerve tissue structure and function. Ideally, a nerve tissue scaffold would serve as a carrier of seed cells and active factors while also filling the lesion site, assisting seed cell survival and proliferation, promoting the reconnection of damaged spinal cord tissue, helping to bridge the gap in the lesion site and rebuild nerve conduction, and facilitating sustained drug delivery. The scaffolds most used for nerve repair include hydrogels, nanoparticles, and nanofibers (Ahmad et al., 2022). In this paper, we describe the characteristics of hydrogels and discuss their applications in SCI repair.

## 3 Characteristics of Hydrogels

**FIGURE 3:**
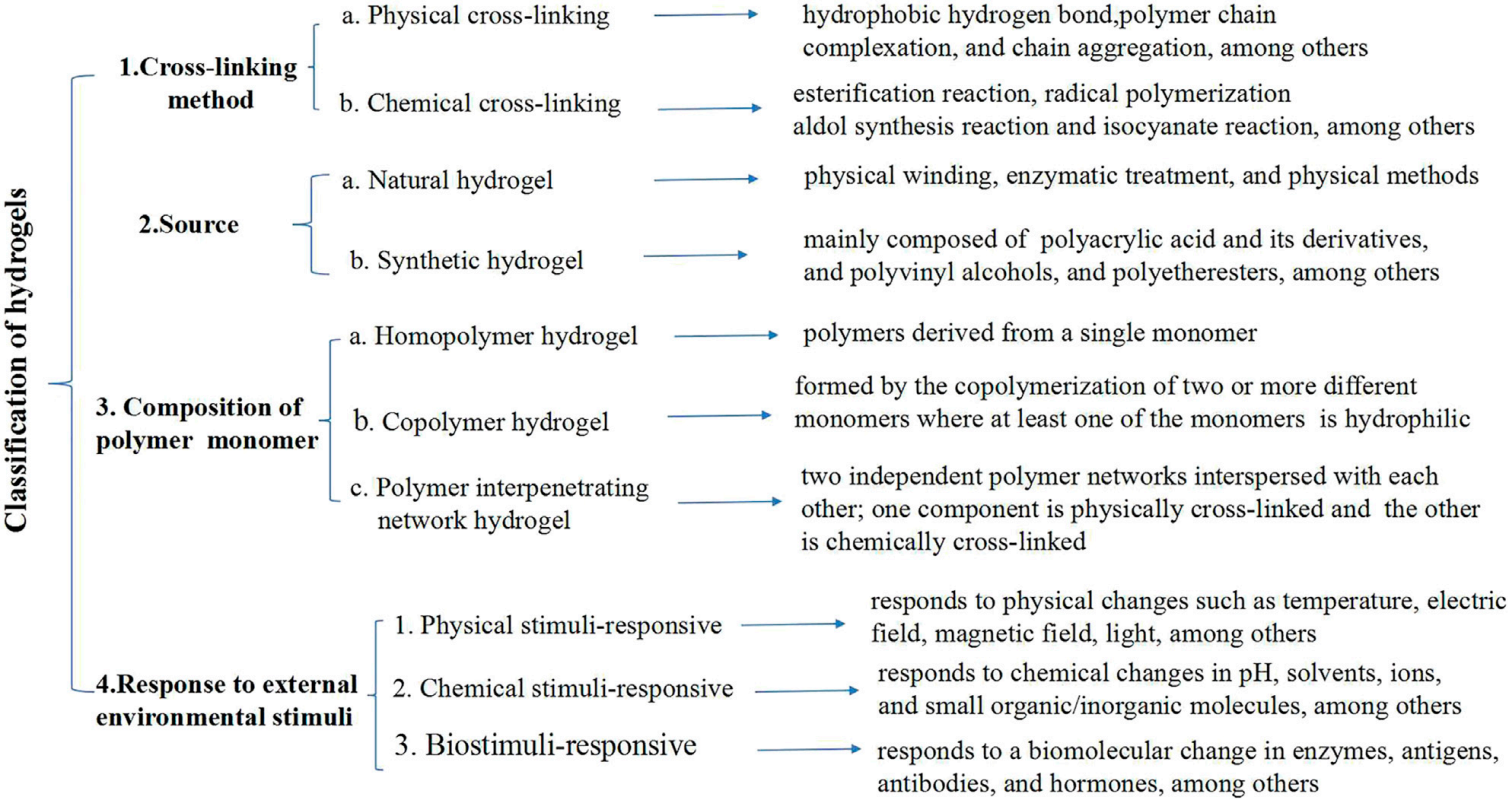
Classification of hydrogels.

Hydrogels are macromolecular porous polymer network systems with a water content higher than 95%. They have viscoelasticity, high flexibility, excellent mechanical properties, strong plasticity, and good biocompatibility and biodegradability. Hydrogels can simulate the extracellular matrix environment and support the exchange of nutrients and surrounding tissues (Führmann et al., 2016). The implantation of hydrogels into injured spinal cord tissue can provide mechanical support for cells and tissues, promote cell migration, and facilitate the long-term controlled release of cellular molecules and drugs (by bridging the gap between the lesions or by providing a platform for the accumulation of neurotrophic factors in the lesion area), thereby mediating spinal cord tissue repair and regeneration (Jain et al., 2006; Toh and Loh, 2014; Assunção-Silva et al., 2015). Hydrogels can be synthesized using physical or chemical methods (Figure 3). Physical hydrogels are cross-linked to form a network structure under the action of non-covalent bonds. Cross-linking pathways include hydrophobic, ionic, electrostatic, and host-guest interactions, as well as hydrogen bonding and phase transitions. Physically cross-linked hydrogels will change with external conditions (e.g., temperature and pH). The preparation conditions for physically cross-linked hydrogels are relatively mild, with dynamic reversibility, self-healing, processability, and repeatability (Liu et al., 2018). Chemical hydrogels are cross-linked through chemical bonding between polymer chains, usually driven by small molecular initiators and external energy such as light and heat. Chemical cross-linking pathways include Michael addition reactions, condensation reactions, cross-linking with aldehydes, Schiff base reactions, thiol-disulfide bond exchange, free radical polymerization, photo-cross-linking, and enzyme-mediated cross-linking. Chemical hydrogels are more stable than physically cross-linked hydrogels; moreover, their structure and properties can be more precisely controlled (Hu et al., 2019). The hardness, morphology, structure, and biochemical modifications of the hydrogel materials will affect cell growth, adhesion, axon growth, proliferation, differentiation, and migration. The raw materials for synthesizing natural gels are usually proteins and polysaccharides. Protein materials are subjected to physical or enzymatic treatments to form hydrogels, while polysaccharides such as agar and chitosan are generally synthesized *via* physical entanglement and cross-linking (Wei et al., 2015).

The uniform pores and parallel arrangement of the scaffold provide guiding channels for cells. If the pores are smaller than the size of the drug or cell, the drug and cell will be encapsulated in the hydrogel and not be released. If the pores are bigger than the drug and cell, the latter will move freely in the hydrogel network for subsequent release (Li and Mooney, 2016; Chen et al., 2018; Trombino and Cassano, 2020; Shoukat et al., 2021). Hydrogels can prevent inflammatory responses and nerve compression, as well as play an important role in controlling the release of molecules, owing to their excellent biodegradability. Controlling the degradation of hydrogels has a strong impact on improving drug and cell release; moreover, the physical and mechanical properties of the hydrogel surface will also affect cell adhesion, differentiation, and axonal growth (Lakard et al., 2004; Zhan, 2020). The smoothness and convexity of the scaffold surface can affect protein expression (Johnson et al., 2018). Animal experiments have shown that the scaffold morphology can promote cell migration and axon regeneration (Chen et al., 2020). Currently, most research on SCI repair is focused on modifying traditional hydrogels, and efforts to further strengthen the therapeutic effect of hydrogels, promote the regeneration of nerve stumps, and rebuild neural circuits should focus on modifying traditional hydrogels and developing new hydrogels with optimized mechanical properties, drug encapsulation and sustained release activities, and the ability to load cellular molecules and drugs (Xu et al., 2013; Ucar et al., 2021; Yao et al., 2021). These studies can be broadly categorized by the composition of hydrogel-based interventions for SCI, namely hydrogel therapy alone, cell-laden hydrogel therapy, drug-carrying hydrogel, or combination therapy with additional factors.

## 4 Hydrogel Therapy

Hydrogels are obtained from numerous sources. They are easy to process, highly flexible, and easily molded (Stegemann and Nerem, 2003). Hydrogels can carry cells in two ways. One is *via* direct injection or implantation at the site of the SCIs, filling the cavities of spinal cord lesions, bridging injury defects, and providing contact guidance for axon regeneration while self-degrading (Piantanida et al., 2019). Hydrogels can also be used as a carrier of transplanted cells or encapsulated neurotrophic factors and other drugs to complete the local enrichment and sustained release of seed cells and drugs so that the loaded molecules can be accurately delivered to the spinal cord, providing a suitable microenvironment for neural reconstruction (Liu and García, 2016; Huang et al., 2017; Dimatteo et al., 2018; Ucar et al., 2021; Yao et al., 2021). One characteristic of this approach is that the strength of the hydrogel and the degradation rate are closely related to cell survival and proliferation. The second approach involves planting seed cells after pre-preparing the scaffold, which has a specific internal structure and geometric shape, as well as the pore size, porosity, and mechanical strength. The disadvantage of this strategy is that the cells cannot be distributed uniformly or permanently in the scaffold space. In recent years, continuous advances in biomedical technology have resulted in the development of smart gels, including pH-sensitive, molecular self-loading, temperature-sensitive, and conductive hydrogels (Dadsetan et al., 2010).

### 4.1 Hydrogel Therapy Alone

**FIGURE 4:**
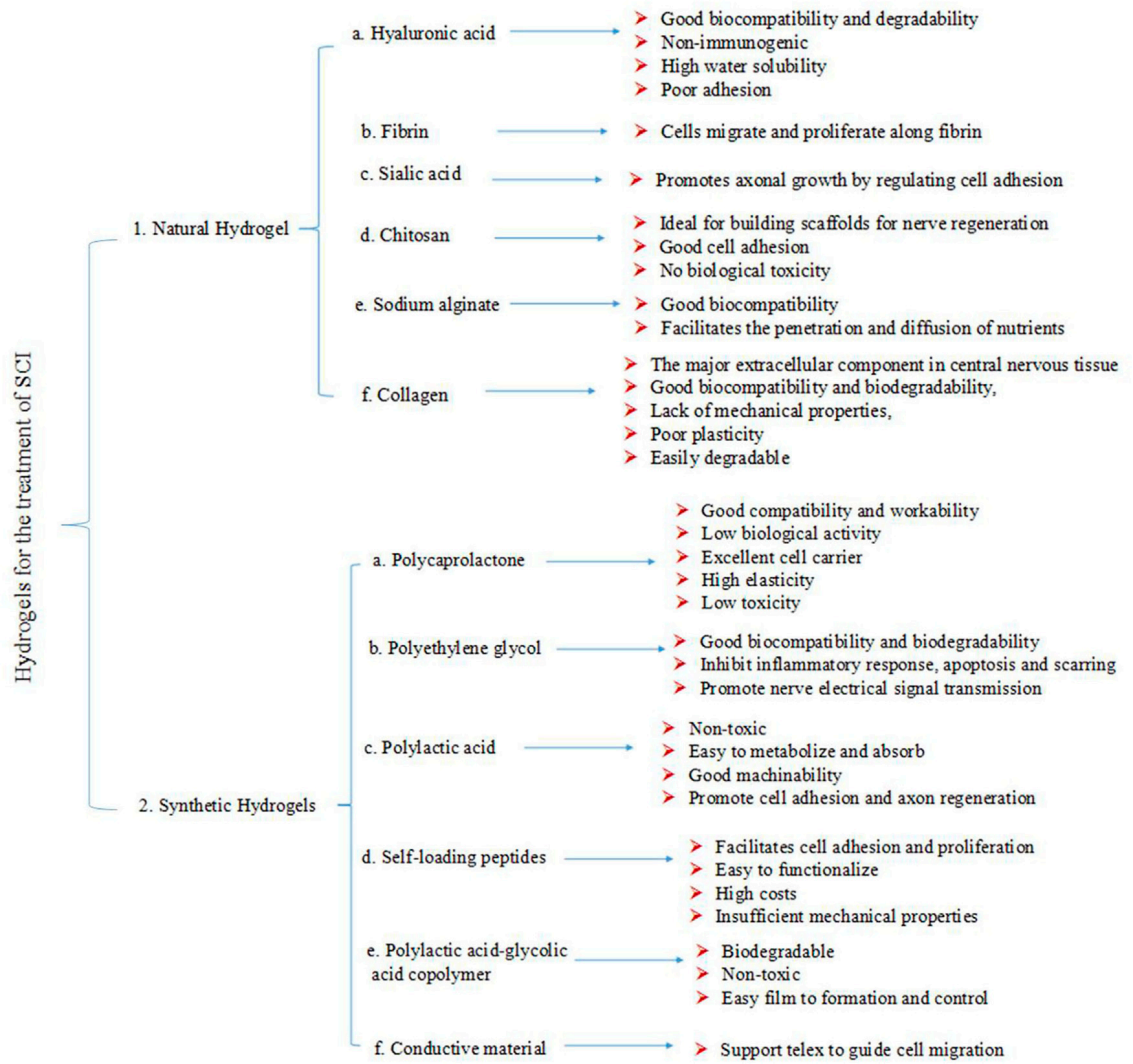
Application and characteristics of hydrogels therapy.

Several studies have described the use of hydrogels, mainly collagen, gelatin, hyaluronic acid (HA), fibrin, chitosan, silk protein, alginate, laminin, agarose, dextran, fibronectin (FN), and their complexes for SCI repair (Hejcl et al., 2008; Gros et al., 2010; Jukes et al., 2010; King et al., 2010; Collins and Birkinshaw, 2013; Mothe et al., 2013; Meng et al., 2014) (Figure 4).

Collagen is the main extracellular component in the central nervous system (CNS). The cell adhesion signal peptide sequence arginine-glycine-aspartic acid (RGD) can guide cells to recognize the scaffold material, which helps maintain the cell’s phenotype and activity (Hosseinkhani et al., 2013). *In vitro* experiments have shown that collagen can promote the proliferation and differentiation of nerve cells and inhibit the proliferation of neurocollagen gliosis (O’Connor et al., 2001; Ma et al., 2004). Three-dimensional collagen hydrogel scaffolds can maintain the self-renewal capacity of neural stem cells (NSCs) through the REDD1-mTOR signaling pathway (Zhang et al., 2016) and affect the differentiation of NSCs via the miR-7-Klf4 signaling pathway (Katz and Burdick, 2009). Researchers have used collagen hydrogels to repair SCI in mice and have shown that liquid collagen injected directly into the spinal cord could quickly gel and form a continuous interface in the spinal cord after inhibition and transection injury (Marchand and Woerly, 1990; Marchand et al., 1993). Furthermore, implanted collagen hydrogels could promote the migration of nerve cells, the growth and regeneration of nerve axons, and the secretion of brain-derived neurotrophic factor (BDNF) and neurotrophin-3 (NT-3) (Marchand and Woerly, 1990; Marchand et al., 1993; Yoshii et al., 2004; Klapka and Müller, 2006; Yang et al., 2010b; King et al., 2010). Collagen and chondroitin sulfate or carbodiimide composites can promote axonal regeneration in SCI (Marchand et al., 1993). In addition, the arrangement of collagen fibers is important to guide cell orientation and migration. The direction of collagen fibrils can be controlled using magnetic nanoparticles and by applying an external magnetic field (Vrana et al., 2007).

Sodium alginate hydrogel displays good biocompatibility and is often used as an injectable carrier to load drugs or cytokines targeted to the injury site (Perets et al., 2003; Grulova et al., 2015). It can be cross-linked in the presence of cations to form a reticulated alginate ion gel that promotes the osmotic diffusion of nutrients and provides three-dimensional growth space for cells (Arlov and Skjak-Braek, 2017). Sodium alginate hydrogels show better mechanical properties and cell viability and are more suitable as scaffolds for neural tissue engineering than sodium alginate + HA or sodium alginate + FN composite hydrogels (Bozza et al., 2014). Suzuki (Suzuki et al., 2002) implanted an alginate sponge into a rat SCI model. At 21 weeks after the operation, several fibers appeared at the injury site, and axons regenerated and formed functional synapses. Covalently cross-linked and lyophilized porous alginate gel inhibits SCI and reactive gliogenesis, as well as promotes axonal growth (Kataoka et al., 2004).

HA is the main component of the extracellular matrix of the CNS. It can reduce inflammatory injury and inhibit the formation of fibrous scars after SCI, improve nerve function recovery following injury as well as promote angiogenesis (Meng et al., 2014). HA is often combined with other materials to construct a composite hydrogel for application in neural tissue engineering, thereby optimizing HA adhesion to cells. For example, Horn used thiol-modified HA cross-linking for rat SCI repair and observed axonal regeneration (Horn et al., 2007). James et al. (Austin et al., 2012) injected a hyaluronan-methylcellulose (HAMC) hydrogel into a chronic SCI model 24 h after the latter was established. The results showed that the submembranous space gelled effectively, reduced glial fibrosis and inflammatory responses in the injured area, and promoted the recovery of axonal transmission and neural function.

Chitosan is a degradable cationic polysaccharide with excellent biocompatibility and low immune rejection. Chitosan degradation products can be metabolized (Martins et al., 2014) and easily made into hydrogels (Delmar and Bianco-Peled, 2016). Chitosan and other polymers are widely used in nerve tissue regeneration (Fang and Song, 2019). Several research groups have used chitosan catheter stents to conduct SCI *in vivo* repair experiments. Chedly et al. (2017) implanted a chitosan hydrogel into a rat spinal cord bilateral hemi-transection model; the chitosan hydrogel promoted the reconstruction of spinal cord tissue and blood vessels, reduced fibroglial scarring, regenerated numerous axons, and modulated the inflammation. Three-dimensional agarose with varying mechanical strengths has different axonal growth-promoting abilities on the dorsal root ganglion (DRG) (Balgude et al., 2001). Agarose is produced using the directional freezing method to make directional through holes, and the regeneration of injured spinal cord axons can be induced along these holes (Stokols and Tuszynski, 2004; Stokols and Tuszynski, 2006).

FN participates in cell adhesion by binding to cell surface receptors and guiding cell differentiation, among other functions. FN has neuroprotective effects against the regulation and amelioration of ischemia-induced necrosis after CNS injury (Meng et al., 2014). As a cell carrier, it can greatly improve the survival rate of seed cells and help achieve a more uniform distribution of cells (Meng et al., 2014). Nazari et al. (2020) confirmed that FN hydrogel scaffolds could improve cell viability, induce stem cells to differentiate into oligodendrocyte precursor cells and promote SCI repair. FN hydrogel also inhibits the recruitment of reactive glial cells in the subacute phase of SCI (Johnson et al., 2010). *In vitro* studies have shown that a composite hydrogel of FN and fibrin (FB) was more effective at promoting axon growth than the single-component FN hydrogel (Meng et al., 2014).

Some synthetic hydrogels also have excellent effects on SCI repair. The polyethylene glycol (PEG) microgel scaffold can be used to delivery and secrete human bone marrow mesenchymal stem cells, and promote the repair of nerve damage (Borgens et al., 2002; Duerstock and Borgens, 2002; Laverty et al., 2004; Caldwell et al., 2020). Wang et al. (2017) prepared polymer micelles-DA/mPEG-PCL scaffolds and applied them in a spinal cord hemi-transection injury model. The results showed that the scaffold could reduce the formation of scars and cysts, as well as promote axon regeneration. In a mouse spinal cord hemisection model, conductive hydrogels (EHs) based on TA and polystyrene (PPv) can promote NSCs differentiation into neurons *in vitro*, and inhibit astrocyte differentiation. *In vivo* results demonstrated that this hydrogel promotes SCI mouseendogenous neurogenesis and functional recovery by restoring interrupted spinal cord circuits. PCL can mimic the structure of gray or white matter and promote the differentiation of NSCs to oligodendrocytes and the formation of axonal myelin sheaths, it is an ideal material in SCI tissue engineering (Donoghue et al., 2013; Patel et al., 2019). Wong et al. (2008) implanted PCL into an animal model of total transection of SCI, and its open microchannel structure facilitates axon regeneration and myelination. Babaloo et al. (2019) combined PCL with gelatin to improve cell adhesion and material degradation rate, as well as promote neuronal and myelin regeneration. The starch-PCL 3D composite scaffold can protected the injured area and promoted the recovery of behavioral function (Silva et al., 2013). Han et al. (2020) prepared an injectable hydrogel containing ursodicholic acid to inhibit inflammation, resist apoptosis, and promote the recovery of nerve function. PLLA scaffolds promoted cell migration, axon regeneration and integration with surrounding host tissues (Deng et al., 2006). Sun et al. (2020) used PLLA nanofiber multi-channel scaffolds filled with NT3-loaded gelatin sponge transplanted into a model of total transection of SCI, which reduced the deposition of collagen fibers and promoted the recovery of animal behavioral function.

### 4.2 Cell-Laden Hydrogel Therapy

**FIGURE 5:**
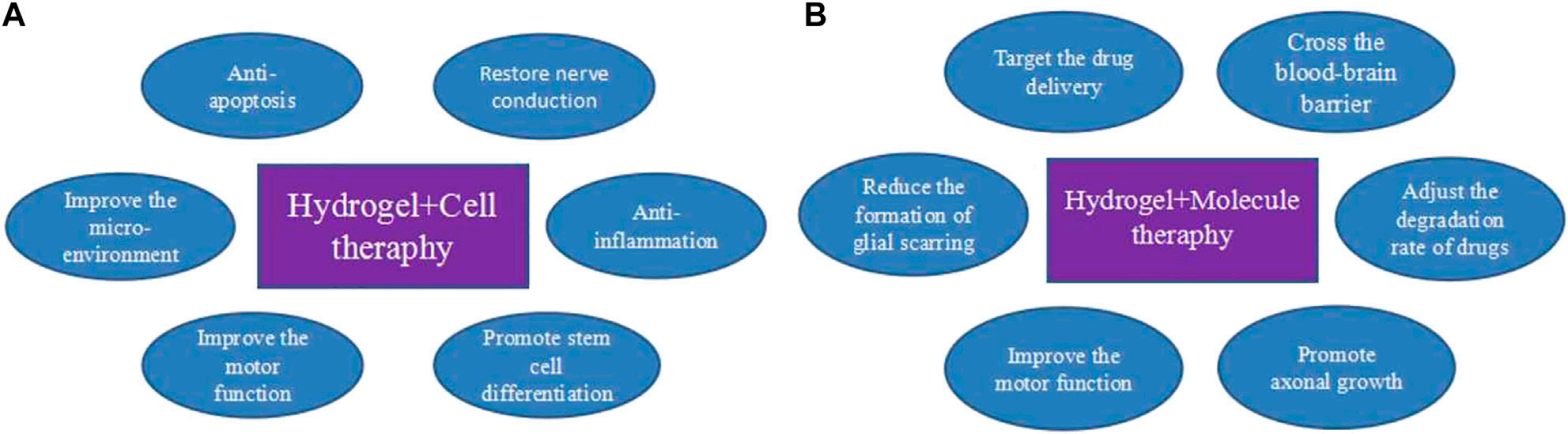
Advantages of hydrogels in the treatment of SCI. **(A)** Hydrogel combined with cell therapy. **(B)** Hydrogel combined with molecular therapy.

The loss of neuronal tissue and the existence of cavities at the injury site make cell transplantation an effective approach to treating SCIs. The seed cells used for SCIs repair mainly include olfactory ensheathing, Schwann, neural stem (Barbour et al., 2013), embryonic stem, induced pluripotent stem, and mesenchymal stem cells (MSCs). The transplanted stem cells can differentiate into neuronal cells, and glial cells secrete various cytokines, inhibit inflammation and apoptosis, promote axon regeneration, and restore interneuron communication (Barbour et al., 2013; Tabakow et al., 2013; Doulames and Plant, 2016; Yang C. et al., 2019; Chen et al., 2019; Chu et al., 2019; Gao et al., 2020). However, single-cell therapy cannot keep the sustained concentration at the damaged spinal cord and use the combined hydrogel and cell delivery system to provide a protection system for cells and avoid apoptosis or necrosis (Figure 5A).

#### 4.2.1 Hydrogel + Neural Stem Cells

Transplanting a mixture of hydrogel and NSCs into injured spinal cord tissue can promote differentiation into neurons, replacing dead neurons and restoring nerve conduction (Chen et al., 2010). These transplanted NSCs can also secrete various neurotrophic factors and cytokines to protect damaged nerve cells, inhibit the inflammatory response, reduce the degree of demyelination of damaged axons, and restore motor function. DNA hydrogel is a three-dimensional network structure formed by high cross-linking of DNA strands in an aqueous solution. It has shape plasticity, excellent biocompatibility and biodegradability, and accurate molecular identification. The network structure of DNA hydrogels facilitates the encapsulation of proteins and cells and regulates the release of these molecules (Patel et al., 2019; Khajouei et al., 2020; Pedersen et al., 2020). Yuan et al. (2021) loaded NSCs with DNA supramolecular hydrogel materials and injected them into the lesions of a rat SCI model with a 2-mm total transaction. The results of the study showed that 8 weeks after transplantation, the motor function of both hindlimbs in SCI rats was significantly improved, voluntary urination behavior was restored, and implanted. NSCs differentiated into neurons, oligodendrocytes, and astrocytes, achieving remyelination, forming new neural circuits, and restoring the signal transmission. Mothe et al. (2013) applied a HAMC gel encapsulated with the blood-derived growth factor rPDGF-A to load NSCs to treat SCI mice. The group receiving the combined treatment of gel and NSCs showed improved survival of neurons and oligodendrocytes in the surrounding tissue. The number of nerve cells increased, the injured area of the mice decreased, and the motor function of the lower limbs recovered better. Collagen and NSCs can form neural networks *in vitro* (Ma et al., 2004). Egawa et al. (2011) used collagen hydrogel as a carrier for NSCs. Polypeptide constructs prepared by recombinant DNA technology bind to collagen to anchor epidermal growth factor (EGF) under mild conditions, stabilizing exogenous EGF in collagen. *In vitro* experiments showed that the EGF-bound collagen hydrogels have significantly increased the number of nerve cells. Yuan et al. (2014) prepared double-layer porous collagen membranes with different pore sizes as the transplant carrier for NSCs. The inner membrane layer had a large pore size that could carry many NSCs, and the outer layer structure could reduce the extension of other surrounding cells into the damaged regenerative microenvironment and glial scar, preventing platelet deposition and external compression at the injury site. This membrane design can significantly promote the differentiation of NSCs into neurons after spinal cord hemi-transection transplantation, improve the regenerative microenvironment, and facilitate the recovery of motor nerve function. The hydrogel scaffold can simulate the electrical conduction characteristics of the natural spinal cord, promoting the differentiation of NSCs and the secretion of related trophic factors (Staples et al., 2017). Hydrogels with higher conductivity can guide NSCs to promote neuron differentiation *in vitro* while inhibiting astrocyte differentiation (Zhou et al., 2018). Single-channel chitosan conduits were filled with neural stem/precursor cells (NSPCs) and photo-crosslinked chitosan hydrogel-growth factor complexes to induce stem cells to differentiate into desired cell types (Li et al., 2014).

Zarei-Kheirabadi et al. (2020) used thiol-modified HA and gel-made hydrogels to encapsulate sorted embryonic stem cell-derived NSCs, which can increase their differentiation into oligodendrocytes and reduce glial scarring, improving motor function in a mouse contusion model. *In vivo* experiments showed that chitosan tubes of composite NSCs/NSPCs could induce NSCs to differentiate into neurons and successfully connect both ends of the injured spinal cord. Sun et al. (2017) showed that IKVAV-modified silk fibroin hydrogels could promote the proliferation and differentiation of NSCs, suggesting that the hydrogels have excellent nutrient transport and signal molecule transport capabilities. Another study reported that NSC proliferation in sodium alginate hydrogels decreases with increasing mechanical properties (Banerjee et al., 2009). The degradation of the hydrogel also promotes NSC as the degraded material provides cells with more oxygen and nutrients (Ashton et al., 2007).

#### 4.4.2 Hydrogel + Mesenchymal Stem Cells

Mesenchymal stem cells (MSCs) are derived from various sources. They have multi-directional differentiation potential and are easy to culture and expand *in vitro* (Parekkadan and Milwid, 2010). MSCs can migrate (chemotactic) to the injured area (Shi et al., 2012) and are ideal seed cells for tissue engineering. Syková et al. (2006) prepared macroporous hydrogels based on 2-hydroxyethyl methacrylate (HEMA) or 2-hydroxypropylmethacrylamide (HPMA) derivatives that covalently bonded with the hydrolytically degradable cross-linker N, O-dimethyl acrylamide hydroxylamide or that were modified by different surface charges, and the hydrogels carrying BM-MSCs. The gel was implanted into the spinal cord hemisection of rats, and the results showed hydrogel system loaded with BM-MSCs promoted axonal growth. Yao et al. (2020) showed that BMSCs loaded with fibrin gel could significantly improve motor function in rats. Yao et al. (2021) used a dual-enzymatically cross-linked gelatin hydrogel with hydrogen horseradish peroxidase (HRP) and galactose oxidase (GalOx) to build biocompatible HUC-MSC-loaded injectable scaffolds that showed a similar modulus to neural tissue thereby improving the survival, proliferation, and differentiation of HUC-MSCs *in vitro*. In addition, HUC-MSC-loaded hydrogels can significantly promote the recovery of hindlimb motor function in SCI mice, inhibit inflammation and apoptosis, and accelerate the repair of damaged spinal cords. Oliveira et al. (2017) showed that gellan gum, collagen, and laminin epitope-rich hydrogels all support adipose-derived mesenchymal stem cell-mediated axonal growth. The viscous hydrogel loaded with MSC-derived exosomes positively affected the efficient recovery of neural tissue (Li et al., 2020). Inoculation of fibrin gel-loaded BMSCs in a rat model of total spinal cord resection can significantly improve motor function in rats. Methacryloyl gelatin (GelMA) is synthesized from methacrylic acid *via* gelatinization. Photoinitiation occurs by introducing phenyl-2,4,6-trimethyl-benzoyl phosphonate (LAP) into GelMA. After preparation, GelMA can be quickly cross-linked and cured under blue light (at a 405-nm wavelength). The production cost of this material is low. Studies have shown that the GelMA concentration affects its pore size, swelling mechanics, and other related physical properties (Bertlein et al., 2017; Celikkin et al., 2018). GelMA hydrogels have good biocompatibility (Fang et al., 2016; Yang D. et al., 2019) and promote BMSC growth. In addition, different concentrations of GelMA have varying effects on the cell growth state (Nichol et al., 2010)—caused by the GelMA concentration changing the degree of cross-linking in photocured GelMA hydrogels (Ren et al., 2018). Zhao et al. (2016) pointed out that the proliferation of BMSCs in photocured GelMA hydrogels might be related to pore size. Zhang et al. (2020) constructed a three-dimensional hydrogel based on HA and modified it with the laminin polypeptide PPFMLLLKGSTR. The hydrogel was loaded with human placental amniotic membrane-derived MSC exosomes and transplanted into a rat SCI model. Twenty-eight days after transplantation, the treatment of hydrogel combined with exosomes significantly promoted the motor function recovery and significantly increased the effective step rate of the rat’s hindlimb. Magnetic resonance imaging showed that the fracture area at the spinal cord was significantly reduced in the hydrogel-exosome combined treatment group relative to the hydrogel-only group. Alginate hydrogels were loaded with Schwann cells combined with BDNF to treat the animal model of C5 semi-transected SCI. Schwann cells and BDNF promoted the regeneration of intact axons across the lesion site to the injured segment (Liu et al., 2017).

#### 4.2.3 Hydrogel + Embryonic Stem Cells

An injectable composite hydrogel system was developed based on a modified gelatin matrix combined with shape memory polymer fibers. This system was used to load ESC-derived motor nerves (MNs) in a directed embryonic stem cells (ESCs) differentiation and minimally invasive manner, then used to treat SCI (Wang et al., 2018). Composite hydrogels with aligned fibers enhanced cell viability and neurite outgrowth *in vitro* and enhanced ESCs differentiation into MNs *in vivo*. ESCs-loaded composite hydrogels transplanted into SCI mice *via* injection significantly enhanced tissue regeneration and angiogenesis, improved the expression of Tuj1, MAP2, and Syn, and decreased immune responses, which recovered motor function in the mice.

#### 4.2.4 Hydrogel + Induced Pluripotent Stem Cells

A dual-porous laminin-coated hydrogel seeded with induced pluripotent stem cell-derived neural progenitor cells (iPSC-NPs) in a rat model of chronic SCI was found to reduce cavitation and support iPSC-NPs survival; however, its use did not lead to a significant improvement in motor recovery (Joung et al., 2018; Ruzicka et al., 2019). Joung et al. (2018) used three-dimensional printing of iPSC-derived spinal neuronal progenitor cells (sNPCs) and oligodendrocyte progenitor cells (OPCs), and the results showed that biomarked sNPCs differentiate and extend axons throughout the microscaffold channels. Bahareh et al. (Nazari et al., 2020) determined the potential of fibrin hydrogels to enhance the differentiation of human fibroblast-induced pluripotent stem cells into oligodendrocytes. iPSCs in HA and gelatin methacrylate-based hydrogels exhibited stronger neurite elongation and spontaneously favorable neuronal differentiation (Wu et al., 2017; Fan et al., 2018). The above results confirm that iPSCs combined with hydrogels can be used to treat SCI.

### 4.3 Molecular-Carrying Hydrogel Therapy

Ordinary hydrogel as a drug delivery system mainly relies on the diffusion of the drug or the degradation of the material itself for release, which is uncontrollable and may not achieve the desired effect of drug delivery (Shoukat et al., 2021). Many drugs fail to accumulate in the CNS after systemic administration because of their inability to cross the tightly regulated blood-spinal cord barrier (Pardridge, 2011). Therefore, there is need for effective, targeted, long-term, and safe approaches for the sustained release of therapeutic substances to improve the restorative effect of SCI (Paves and Saarma, 1997; Ramer et al., 2000; La Manna et al., 2021). Drug delivery system (DDS) can effectively improve the therapeutic effect of drugs, reduce drug toxicity and improve patients’ dependence on drugs. Ideally, the DDS must have good biocompatibility and must protect the drug by avoiding degradation while maintaining its stability and activity (Lagreca et al., 2020; Yang J. et al., 2021; La Manna et al., 2021). After injecting the DDS into the affected cord region, hydrogels quickly convert from liquid to gel at the injury site, bridging small gaps between the spinal cord tissue (Figure 5B).

#### 4.3.1 Hydrogel + Chemical Drug

Hydrogels can encapsulate drugs or drug-loaded carriers to provide sustained local drug release. Additionally, factors including the preparation method, structure, shape, pore size pH, light, magnetic field, electric field, ionic strength, and enzymatic environment of the hydrogel are closely related to drug loading and release capacity (Günther et al., 2015; Barclay et al., 2019). Local delivery can be achieved by injecting preformed hydrogels or polymer solutions gelled *in situ* into the parenchymal, intrathecal (subdural), or epidural space (Shoichet et al., 2007). Burdick et al. (2006) noted slower drug release from gels with high cross-link density, and Lindsey et al. (2015) showed that drug or growth factor release rates were inversely proportional to the hydrogel concentration. Caccavo et al. (2015) found that as the effective pore size increases during the degradation process, the drug release rate can be manipulated using varying hydrogel degradation rates. In addition, the drug and the hydrogel necklace are linked by degradable covalent bonds, and the release system can be regulated by adjusting the ratio of the drug to the binding site and the affinity between the drug and the hydrogel.

Li et al. (2018) loaded paclitaxel-encapsulated liposomes into collagen microchannel scaffolds, which prolonged the sustained release time of paclitaxel. Fingolimod promotes NSC proliferation and induces the favorable differentiation of NSCs, thereby promoting remyelination. It is used in bioscaffolds to be co-implanted with NSCs in SCI lesions (Wang et al., 2015). Injectable hydrogels represented by thermosensitive hydrogels are suitable for treating nerve repair because their injection can minimize the damage to the injured area, fill the cavity generated by the lesion and ensure that the drug is carried directly into the lesion site (Panzer et al., 2020). Zhang et al. (2018) use dpolysialic acid (PSA) to control the developmental properties of the CNS by regulating cell adhesion and promoting axonal growth. They prepared a PSA/PCL scaffold to encapsulate methylprednisolone (MP). The composite scaffold was transplanted into a rat SCI model, and the findings showed that PCL/PSA/MP with MP could inhibit the expression of axonal demyelination and glial fibrillary acidic protein but promote the recovery of motor function (Zhang et al., 2018).

#### 4.3.2 Hydrogel + Growth Factor

Neurotrophic factors can promote neuronal survival and axonal growth during embryonic development, protect damaged neurons and promote the proliferation and differentiation of NSCs (Zhang et al., 2016; Zweckberger et al., 2016). Moreover, they play an important role in preventing microenvironment-induced secondary injury. Many studies have focused on loading neurotrophic factors into bioscaffolds to improve the safety and targeting of damaged nerve repair (Perale et al., 2011). This method helps growth factors overcome the difficulty of passing through the blood-brain barrier and the risks associated with long-term administration (Bajaj et al., 2014; Kondiah et al., 2016). Dai’s team used coaxially arranged collagen fiber bundles to functionally bind factors and drugs such as NT3, BDNF, bFGF, LDN193189, SB431542, CHIR99021, P7C3-A20, Eph A4LBD and Plexin B1LBD, and loaded NSCs to repair SCI, significantly reducing the cavitation in the injured area, attenuating the reactive proliferation of glial cells, and promoting axon regeneration and myelination (Han et al., 2019; Liu W. et al., 2020; Yang Y. et al., 2021). Collagen gel complexed with NT-3 can promote axon regeneration in the spinal cord and restore certain functions (Houweling et al., 1998). The copolymer gel that collagen adsorbs NT-3 and FGF-1 to fill poly hydroxyethyl methacrylate, and methyl methacrylate (pHEMA-MMA) promotes the repair of whole SCI in rats (Dewitt et al., 2009). Taylor et al. (2006) implanted a fibrin-loaded NT-3 scaffold into a rat SCI model and reported that fibrin scaffold implantation could significantly reduce glial scarring at the white matter margins of lesions. GDNF was encapsulated in microspheres and delivered to the SCI site *via in situ* injections of sodium alginate gel. The release rate slowed in the rat spinal cord hemi-transaction model; the hydrogel-loaded animals had better functional recovery than the non-GDNF hydrogel-treated group (Ansorena et al., 2013). Zhao et al. (2017) synthesized a new type of heparin-poloxamer (HP) hydrogel, i.e., GDNF bound with temperature-sensitive HP hydrogel. *In situ* injections of GDNF-HP to the injured spinal cord had beneficial effects, including promoting GDNF on the proliferation of NSCs, inhibition of reactive astrogliosis, axon regeneration, neuroprotection against apoptosis, and recovery of bodily functions. Grous et al. (2013) used poly isopropyl acrylamide (PNIPAAAm) and polyethylene glycol (PEG)-cross-linked composite hydrogels to successfully transplant cells or BDNF into the SCI and promote axonal growth and myelination. One-armed or branched PEG is often used as a cross-linking agent to prepare different hydrogel materials (e.g., PEG-cross-linked HA and gelatin composite hydrogels) for transplantating of OPCs into SCI, which can promote their survival and remyelination. Heparin hydrogel was combined with neuroprotective factor (FGF4) to treat SCI in a rat model. The hydrogel released FGF4 in the injured area, which helped inhibit the inflammatory response, increase remyelination, and reduce glial scarring (Wang et al., 2019). EGF-enriched hydrogels promoted synaptic plasticity under local hypoxia after SCI *via* downregulated the *Fbln5* and *Rtl-S3* genes, and upregulated *Clcfl*, *Tgml*, and *Ptgs2* genes (Chan et al., 2019).

#### 4.3.3 Hydrogel + Peptide

A new biomaterial, i.e., self-assembled peptide hydrogel has the advantages of moderate mechanical tension and permeability, low cost, good viscoelasticity, good biocompatibility, low immunogenicity, and being non-inflammatory (Rad-Malekshahi et al., 2016). The degradation products of self-assembled peptide hydrogels can be absorbed and are utilized widely in tissue engineering, drug release, hemostasis, and as antibacterial agents. Recent studies have shown that self-loading peptides have neuroprotection after SCI during transplantation, reduce glial scar formation and damage cavity formation, and promote nerve repair.

The chemical properties of hydrogels usually make it difficult to promote cell adhesion and new tissue formation. Certain chemical modifications are usually used for these non-cell-adhesive hydrogels, such as adding natural extracellular matrix components to modulate cell adhesion and growth (Hiraoka et al., 2009). IKVAV-, GYIGSR-, and RGD-modified natural hydrogel materials were shown to promote neural cell adhesion and better direct differentiation (Hiraoka et al., 2009). Agarose hydrogels supplemented with RGD polypeptides can promote cell migration and synapse growth in three-dimensional cultures (Kriebel et al., 2014). Park et al. (2010) designed and synthesized an -IKVAV/-RGD nanofiber hydrogel that can support neural progenitor or neural stem cell differentiation into neurons and astrocytes in a three-dimensional environment. Additionally, this hydrogel provides a more favorable environment for nerve regeneration in sciatic nerve defects, intracerebral hemorrhage, and spinal cord transaction models. Gelain et al. (2012) injected the Ac-FAQ-LDLK12 self-assembled short peptide hydrogel scaffold into an acute SCI model. The results showed that it could promote nerve tissue regeneration and improve motor skills function. Cigognini et al. (2014) and others injected RADA16-4G-BMHP1 and Ac-FAQ-LDLK12 hydrogels into the SCI site. Both short peptides had a hemostatic effect on the third day after the injury, and axon regeneration at the injury and spinal cord hematoma site was significantly improved 28 days after transplantation. Guo et al. (2007) isolated Schwann and nerve cells and implanted them into RADA16 self-assembled short peptide hydrogel scaffolds transplanted into SCI rats. Both Schwann and nerve cells migrated, and spinal cord repair was observed, showing that the self-assembled short peptide hydrogel scaffold played a bridging role in the injured spinal cord tissue. Nanofibrous hydrogel materials linked to IKVAV polypeptides can significantly promote the adhesion of neural cells and the differentiation of stem cells into neurons (Sehgal and Banerjee, 2013). Cheng et al. (2013) found that RADA(16)-IKVAV can self-assemble into a nanofiber morphology with a double-layer β-sheet structure and form a hydrogel with mechanical stiffness similar to that of brain tissue. Histological analysis showed that RADA(16)-IKVAV self-assembled peptide hydrogels enhanced the survival of loaded NSCs and reduced astrocyte generation. Chitosan hydrogel linked to NT-3 enhanced the ability of NSCs to differentiate into neurons (Yang et al., 2010a). Sodium alginate can generate highly active aldehyde groups grafted with various cytokines and peptides (Le-Tien et al., 2004). Liu et al. (2013) injected K2(QL) and 6K2(QL6) hydrogels into SCI rats and found that it could significantly reduce post-traumatic apoptosis, inflammatory response, and astrogliosis, as well as contribute to organizing protective effects. Liu H. et al. (2020) used RADA16-IKVAV for loading CNTF, a FGF, EGF, and PDGF-AA and RADA16-RGD for loading BDNF, NT3, IGF, b FGF, GDNF, and β-NGF. The prepared functional self-assembling polypeptide hydrogel was implanted into the SCI model, promoting the recruitment and proliferation of endogenous NSCs in the injured area and promoting the regeneration of myelinated axons. Iwasaki et al. (2014) also used the self-assembling polypeptide QL6 series to encapsulate neural progenitor cells (NPCs) to improve the microenvironment after SCI. The QL6 series grafts were effective in acute inflammation of SCI. The protection of MNs in areas adjacent to the injury in a mouse model of SCI was significantly better than that of the control group at 12 weeks after transplantation. Additionally, improved neurobehavioral function, as well as increased activity area and step length, were detected in the forelimbs of rats. Zweckberger et al. (2015) injected QL6 self-assembled peptide scaffolds into the injury site 2 weeks after the acute phase of SCI. They injected NPCs into the adjacent spinal cord, combined with a slow-release growth factor micropump to ensure cell survival. The hydrogel matrix could effectively improve the viability and differentiation potential of the cells and reduce scar tissue formation. The self-assembled short-peptide hydrogel SPG-178 scaffold designed by Ando et al. (2016) can induce neurotrophic factors in the treatment of SCI. *In vitro* studies have shown that SPG-178 can increase the levels of the nerve growth factor brain-derived neurotrophic factor (NT-4), TrkA, and TrkB. *In vivo* studies show that SPG-178 can increase the expression of neurotrophic factors, as well as reduce inflammation and glial scarring. Using N-isopropylacrylamide (NIPAM) monomer and PEG as raw materials to synthesize thermosensitive hydrogels with fast temperature response *via* reversible addition-fragmentation chain transfer (RAFT) polymerization while introducing the polypeptide IKVAV, which has specific biological activity on spinal cord repair, improves the material’s biological activity. The constructed gel scaffolds were cultured with NSCs *in vitro*, and the results showed that the gel scaffolds could promote the adhesion of NSCs and the expression of the adhesion gene *Lamb2*, effectively guiding stem cell differentiation into neurons. The results of *in vivo* experiments showed that the motor function of the rats was restored and that the gel scaffold effectively reduced glial scar formation, lesion size, and cyst area (Long et al., 2020).

## 5 Conclusion

SCI is typically caused by vertebral body injury, resulting in neurological or motor dysfunction and placing a heavy burden on patients and their families. Traditional treatment methods do not allow drugs to pass the blood-brain barrier and concentrate effective doses on the injured spinal cord. Hydrogels can effectively simulate the local soft tissue environment of injury. Optimized hydrogels that are modified using different methods can be used as scaffolds to load drugs or cells, achieve local long-term release, support and guide axon regeneration, and effectively improve the treatment of spinal cord injury. They also overcome the uncertainty of local drug administration, improve the utilization rate of drugs or cells, and enhance the duration of drug action. The models currently used in SCI mainly include the spinal cord contusion model (the most widely used in scientific research), the spinal cord transection injury model, the spinal cord compression injury model (a common model for SCI), the spinal cord distraction injury model, the transverse spinal cord fracture and dislocation (cervical spinal cord injury) model, and the spinal cord ischemia-reperfusion injury model. Each model varies in its degree of damage performance; therefore, the applied treatment cannot fully evaluate the treatment effect. In addition, the repair effect of hydrogels on models of SCI caused by inflammation or tumor cannot be assessed. Axon regeneration is an important evaluation index for SCI repair; however, whether new axons have biological functions similar to the unique nerve tracts in spinal cord tissue requires further investigation. The microenvironment is another important factor in SCI repair. Future work on hydrogels should focus on how to modify their properties, improve their loading capacity, promote cell proliferation and differentiation, and release drugs and factors reasonably and effectively as well as explore more suitable and effective novel hydrogel scaffold materials, construct a combined treatment of hydrogel-carrying cells and drugs, and improve the microenvironment of the injured area to achieve complete repair of SCI.
